# Mesenchymal Stem Cells Improve Cognitive Impairment and Reduce Aβ Deposition *via* Promoting AQP4 Polarity and Relieving Neuroinflammation in Rats With Chronic Hypertension-Induced Cerebral Small-Vessel Disease

**DOI:** 10.3389/fnagi.2022.883503

**Published:** 2022-05-19

**Authors:** Xiao lu Liu, Fu bing Ouyang, Liu ting Hu, Pei Sun, Jing Yang, Yuan jing Sun, Meng shi Liao, Lin fang Lan, Zhong Pei, Yu hua Fan

**Affiliations:** Department of Neurology, The First Affiliated Hospital, Sun Yat-sen University, Guangdong Provincial Key Laboratory of Diagnosis and Treatment of Major Neurological Diseases, National Key Clinical Department and Key Discipline of Neurology, Guangzhou, China

**Keywords:** mesenchymal stem cells, cerebral small-vessel disease, amyloid β-protein, aquaporin 4, neuroinflammation, vascular cognitive impairment, white matter lesions

## Abstract

Cerebral small-vessel disease (CSVD) is the main cause of vascular cognitive impairment (VCI), and the accumulation of amyloid β-protein (Aβ) may be significantly involved in CSVD-induced VCI. The imbalance between Aβ production and clearance is believed to be an important pathological mechanism of Aβ deposition in Alzheimer disease. In this study, we aimed to disclose the roles of aquaporin 4 (AQP4) and neuroinflammation in CSVD, which were the key factors for Aβ clearance and production, respectively, and the effect of mesenchymal stem cells (MSCs) on Aβ deposition and these two factors. The stroke-prone renovascular hypertensive (RHRSP) rats were grouped and received MSC and MSC + AS1517499 (an inhibitor of pSTAT6). The latter was used to explore the underlying mechanism. The cognitive function, white matter lesions, Aβ expression, expression, and polarity of AQP4, neuroinflammation and the STAT6 pathway were investigated. Compared with sham-operated rats, RHRSP rats showed spatial cognitive impairment, white matter lesions and Aβ deposition. Moreover, AQP4 polarity disorder and neuroinflammatory activation were found, which were linked to Aβ deposition. Treatment with MSCs markedly improved cognitive tasks and reduced Aβ deposition but failed to reduce white-matter lesions. Furthermore, MSCs not only promoted AQP4 polarity but also alleviated neuroinflammation probably through the STAT6 pathway. The present study demonstrated that Aβ deposition, AQP4 polarity disorder and neuroinflammation might be involved in CSVD and the regulatory effects of MSCs on them suggested potential therapeutic value for CSVD.

## Introduction

Cerebral small-vessel disease (CSVD) is the clinical, radiological, and pathological syndrome caused by lesions involving cerebral microvessels (Pantoni, 2010), causing various lesions such as white matter hyperintensities (WMH) usually identified by neuroimaging with MRI (Wardlaw et al., 2013; van Veluw et al., 2017). As one of the most important causes of vascular cognitive impairment (VCI) (Inzitari et al., 2009; Gorelick et al., 2011; Hachinski, 2015), CSVD affects almost everyone older than 90 years. Because of its high age correlation, CSVD is related to pathological changes similar to those in neurodegenerative diseases, such as amyloid β-protein (Aβ) deposition (Sweeney et al., 2018). The pathology of vascular Aβ in cerebral amyloid angiopathy (CAA, a subtype of CSVD) coexists with the pathology of Alzheimer disease (AD).

The imbalance between Aβ production and clearance is an important pathological mechanism of Aβ deposition in AD (Hardy and Selkoe, 2002; Selkoe, 2003). In the process of clearance, there has been evidence that soluble metabolites in the brain can be eliminated along the perivascular space (PVS) (Iliff et al., 2013; Abbott et al., 2018). Patients with CSVD often have enlarged perivascular space (EPVS), which was considered likely reflected perivascular clearance disorders. This perivascular pathway that clears interstitial solutes was termed the “glymphatic pathway” for its functional similarity to the peripheral lymphatic system (Iliff et al., 2012; Mestre et al., 2017). It has been found that EPVS is significantly associated with vascular Aβ deposition (Perosa et al., 2022). Moreover, the glymphatic system has been linked to plaque formation in AD (Iliff et al., 2012; Arbel-Ornath et al., 2013; Xie et al., 2013). The arterial pulsation is the main driver of the glymphatic flow, and arteriosclerosis may impede this process. Mortensen et al. (2019) reported that glymphatic clearance was inhibited in stroke-prone spontaneously hypertensive rats (SHRSP), suggesting a new link between vascular pathology and AD. Previous studies also showed that reduced cerebral blood flow increased the deposition of Aβ in patients with AD, which might be caused by glymphatic system disorder that due to the abnormal blood flow (Hama et al., 2015; Saito and Ihara, 2016). The glymphatic system adjacent to the blood vessels is surrounded by the end feet of astrocytes, which are rich in aquaporin 4 (AQP4) (Nedergaard and Goldman, 2016). AQP4 plays an important role in maintaining the function of the glymphatic system. Loss of AQP4 in mice impaired glymphatic clearance of soluble macromolecules (Iliff et al., 2012; Xu et al., 2015). In addition, the improvement in AQP4 polarity promoted the clearance of Aβ by the glymphatic system (He et al., 2017). These studies suggested that astrocyte AQP4 was involved in the process of Aβ clearance, but the existence of related pathological changes in CSVD has not been supported by evidences.

On the other hand, as an important cause of Aβ formation, neuroinflammation has been widely thought to be closely related to chronic degenerative diseases, such as AD and Parkinson disease (Kinney et al., 2018). Also, microglia, in particular, has been proved to trigger the inflammatory cascades in the brain (Gogoleva et al., 2019). Activated microglia has two phenotypes: proinflammatory MI type and anti-inflammatory M2 type. Aβ can induce neuronal oxidative stress, activate microglia to convert into MI type, and trigger neuroinflammation and neuronal apoptosis. At the same time, inflammation may lead to more Aβ deposition, which leads to the transformation of acute injury into chronic injury (Garcia-Alloza et al., 2013; Saito and Saido, 2018). In the chronic cerebral hypoperfusion model, the activation and proliferation of glial cells has been observed to be an important pathological process leading to cognitive impairment (Barrientos et al., 2015; Wang et al., 2019), as well in RHRSP rats (Cai et al., 2020). Therefore, regulating the transformation of microglia into M2 type, an anti-inflammatory phenotype, is a new target for disease treatment. Studies on macrophages revealed that signal transduction and activator of transcription (STAT) family played an important role in microglia or macrophage phenotypic transformation (Sica and Bronte, 2007). STAT1 promoted the transformation of IFN-γ-induced M1 phenotype (Qin et al., 2012), and STAT6 was found to be involved in transforming microglia into beneficial phenotype and functional recovery in MCAO mice (Liu et al., 2016; Cai et al., 2019). Therefore, STAT6 might play an essential role in regulating neuroinflammation induced by cerebral ischemia. However, whether STAT6 is involved in the phenotypic regulation of microglia in CSVD brain lesions remains unclear.

Mesenchymal stem cells are multipotent cells mainly reflected in neuroprotection (Sasaki et al., 2009), neovascularization (Onda et al., 2008), anti-neuro inflammation, and neuroplasticity (Suzuki et al., 2013), supporting the use as a potential therapy to promote functional recovery after stroke and chronic degenerative diseases. Wu et al. found that MSCs could alleviate AQP4-dependent glymphatic clearance in mice with Huntington disease (Wu et al., 2020). MSCs were also found to promote the M2-type transformation of microglia *in vitro* and the expression of anti-inflammatory factors, but the mechanism remains unclear (Gao et al., 2014). Currently, studies on the use of MSCs in CSVD-induced VCI are few. Whether MSCs can improve the pathological changes related to chronic degenerative diseases in CSVD, such as Aβ deposition, and whether STAT6 is involved in the regulation of MSCs on the microglia phenotype in this disease are still unclear.

The present study investigated the cognitive function, Aβ deposition, distribution of AQP4 polarity, and neuroinflammation in RHRSP rats, and the effects of MSCs on cognitive impairment and aforementioned pathological changes, as well as the potential mechanism.

## Materials and Methods

### Experimental Animals

The study was approved by the Animal Research Ethics Committee at Sun Yat-sen University (Guangzhou, China). Male Sprague-Dawley rats (4 weeks old) were purchased from the Laboratory Animal Center of Sun Yat-sen University (Guangzhou, China) and housed under controlled temperature, humidity and a constant light-dark cycle (12-h:12-h). They were also given free access to water and food. All efforts were made to minimize the number and sufferings of animals.

### Study Design

**Figure 1:**
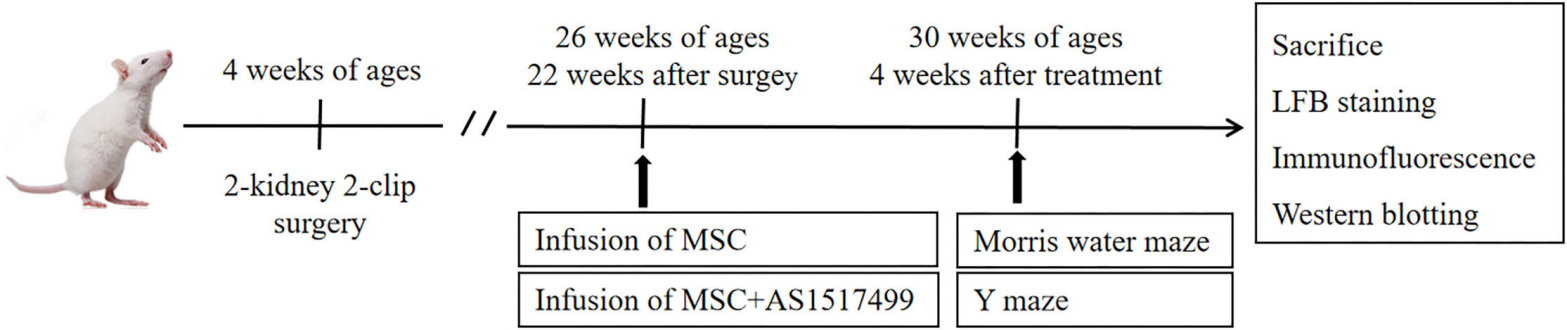
Experimental protocol. Rats received surgery at the age of 4 weeks to establish RHRSP animal model. 22 weeks after the surgery, intervention treatment was applied. Morris water maze and Y maze were performed after 4 weeks of intervention treatment and then rats were sacrificed.

Total 57 rats were randomly divided into sham-operated group (*n* = 12), and operated group (*n* = 45). After the 12 weeks of the two-kidney two-clip operation, the systolic blood pressure (SBP) was measured (tail-cuff sphygmomanometer, BP-2010A, Softron, Japan). The rats with SBP ≥180 mmHg and no obvious stroke symptoms at 22 weeks after surgery were included in the experiment and further randomly divided into RHRSP group (*n* = 15), RHRSP + MSC group (*n* = 15), and RHRSP + MSC + AS1517499 group (*n* = 15). Figure 1 shows the design of the experiments.

### Rat Model of RHRSP

The RHRSP model was established following the two-kidney two-clip method (Zeng et al., 1998). Briefly, 1% pentobarbital (50 mg/kg) was used to anesthetize the animals, and a midline longitudinal incision in the abdomen was made to expose the bilateral kidneys. The renal artery was separated bluntly and silver clips with an inner diameter of 0.3 mm were used to clamp the roots of bilateral renal arteries. The control group received laparotomy without the clamp of clips. The mental state and diet of rats were observed after the surgery.

### Culture of MSCs

Human urine cell-derived induced pluripotent stem cells (U-iPSCs) used in this study were donated by the Guangzhou Institute of Biomedicine and Health, Chinese Academy of Science (Guangzhou, China). The human MSCs were derived from U-iPSCs following a previous study (Gao et al., 2017).

### Intravenous Infusion of MSC/MSC + AS1517499

The RHRSP + MSC group rats were injected with 4.0 × 10^6^ MSCs suspended in 1 ml of sterile phosphate-buffered saline (8mM Na_2_HPO_4_+136mM NaCl+2mM KH_2_PO_4_+2.6mM KCl) *via* the tail vein, and the RHRSP group rats received 1 ml of phosphate-buffered saline without cells. In the RHRSP + MSC + AS1517499 group, the rats were intraperitoneally injected with AS1517499 (10 mg/kg, dissolved in 10% DMSO, 40% PEG300 and 5% Tween80 in saline) 1 h before the intravenous injection of MSCs (4.0 × 10^6^, 1 ml).

### Cognitive Tests

After the 4 weeks of the infusion of MSCs, the Morris water maze test was conducted as described previously (Vorhees and Williams, 2006; Tang et al., 2015). Before one day of the trial, the rats were trained to adapt to swimming freely in the device for 180 s. During the next 5 days of the navigation test, the rats were daily put into the water from four quadrant positions sequentially and given a maximum of 60 s to swim to find and climb the hidden platform. The 6th day was for the spatial probe test. The platform was withdrawn, and a single 60-s swim trial of rats was recorded, which was used to record how many times the rats crossed the hidden platform, the time ratio in the quadrant where the platform was and the initial angle of tracks. The swim paths were recorded with the SuperMaze software (Xinruan Information Technology, Shanghai, China).

In the Y-maze test (Washida et al., 2010), the rats were placed in one of the arms of a 3-arm maze (the angle between any two arms was 120 degrees) to freely move for 8 min. The movement trajectories in the maze were noted, and spontaneous alternation behavior, that is, successive triplet sets, was determined using the following formula: [consecutive entries into three different arms divided by (total number of arms entries minus 2)] × 100. A higher percentage of spontaneous alternation behavior implied improved cognitive function.

### Histological Evaluation of White Matter Lesions

The rats were anesthetized with 1% pentobarbital (50 mg/kg) and perfused transcardially with 0.9% saline followed by 4% formaldehyde. The brains were removed and dehydrated using gradient sucrose and then sectioned at a thickness of 10 μm using a cryostat. Luxol fast blue staining was used to examine myelin loss, and the myelin staining area was used to compare the distribution of myelin in each group. The staining was performed according to standard procedures (Barati et al., 2022).

### Immunofluorescence

Anesthetized rats (1% pentobarbital) underwent the transcardiac perfusion of 0.9% saline, followed by treatment with 4% formaldehyde. The brains were removed and soaked in 4% paraformaldehyde overnight, followed by gradient sucrose dehydration. The coronal brain slices (10 μm) were prepared using a cryostat. The sections were incubated with serum albumin and 0.3% Triton X-100 at room temperature for 1 h. Subsequently, the sections were incubated overnight at 4°C with the following primary antibodies: anti-AQP4(ab9512, 1:200, Abcam, USA), anti-GFAP (ab7260, 1:800, Abcam, USA), anti-GFAP(BM0055, 1:400, Boster, China), anti-Aβ1-42(SIG39142, 1:200, Biolegend, USA), anti-Iba-1 (ab5076, 1:400, Abcam, USA), anti-iNOS (AF0199, 1:200, Affinity, USA), anti-Arg-1 (16001-1-AP, 1:300, Proteintech, USA), anti-C3 (A13283, 1:100, Abclonal, China), and anti-S100A10 (PA5-95505, 1:100, Thermo scientific, USA). The difference between these two GFAP antibodies lies in their different species; they were selectively used when co-stained with different indicators. For example, GFAP (rabbit, Abcam) was used to co-stain with mouse derived AQP4antibody, and GFAP (mouse, Boster) was used to co-stain with rabbit derived C3 antibody. Then, the sections were incubated with appropriate fluorophore-conjugated secondary antibodies for 1 h at room temperature. The immunofluorescence-labeled cells were observed under a fluorescence microscope (Nikon, Tokyo, Japan) and a laser-scanning confocal microscope (Nikon C2).

The fluorescence intensity and the number of cells were quantified following the approaches used previously (Cai et al., 2020). About three separate sections of each rat and eight nonoverlapping 40× fields in the cortex and the hippocampus areas were chosen in a random and blinded manner to quantify the expression of Aβ1-42, AQP4, Iba-1, GFAP, Iba-1/iNOS-positive M1 microglia, Iba-1/Arg-1-positive M2 microglia, GFAP/C3-positive A1 astrocytes and GFAP/S100A10-positive A2 astrocytes. The unit of integrated densities is pixel. The AQP4 polarity, evaluated using a laser scanning confocal microscope with a 60× objective, was calculate during the ratio of the mean intensity of low stringency to high stringency as previously described (Wang et al., 2012). The low-stringency threshold meant the overall area of AQP4 immunofluorescence the high-stringency threshold meant the area of intense AQP4 immunofluorescence localized to perivascular astrocytic end feet in sham-operated rats. Thus, the “AQP4 polarity” was defined as the ratio of the mean intensity of low stringency to high stringency.

### Western Blot Analysis

Western blot analysis was performed following the protocol from previous studies (Fan Y. et al., 2015). The anesthetized rats were transcardially perfused with saline. The brains were removed, and the hippocampi were dissected. Lysis buffer was prepared by RIPA and PMSF according to a ratio of 100:1, and one tablet phosphatase inhibitor (4906837001, Roche, Switzerland) was added to every 10 mL lysis buffer. The tissues were homogenized in lysis buffer (containing phosphatase inhibitor). After cracking and centrifugation, the supernatant was collected. Equal amounts of total proteins were resolved by SDS-PAGE and transferred on to a nitrocellulose membrane. The membranes were incubated overnight at 4°C with the following primary antibodies: anti-STAT6 (ab217998, 1:1,000, Abcam, USA), anti-pSTAT6 (ab263947, 1:1,000, Abcam, USA), anti-TGF-β1 (ab92486, 1:1,000, Abcam, USA), anti-IL-10 (DF6894, 1:500; Affinity, USA), anti-IL-1β (AF5103, 1:500; Affinity, USA), anti-IFN-γ (ab9657, 1:1,000, Abcam, USA), and anti-α-tubulin (2144, 1:1,000, CST, USA). Then, the membranes were washed with 1 × TBST (10 mM Tris-HCl+10 mM NaCl+0.05%Tween-20), incubated with appropriate secondary antibodies: HRP-conjugated anti-rabbit antibody (5571, 1:3,000, CST, USA), at room temperature for 1 h, and then treated with ECL substrate (Millipore, USA).

### Statistical Analysis

All data were expressed as mean ± standard error of the mean (SEM). The Image J software (National Institutes of Health, USA) was used to calculate the immunofluorescence intensity and the relative expression levels of target proteins. IBM SPSS v22.0 (Chicago, IL, USA) and GraphPad Prism 7 software (San Diego, CA, USA) were used to perform one-way analysis of variance followed by the least significant difference *t* test. A *p* < 0.05 indicated a statistically significant difference.

## Results

### Mortality and SBP

Exactly 22 weeks after surgery (before other interventions), 3/15, 2/15, and 3/15 rats died in the RHRSP group, RHRSP + MSC group and RHRSP + MSC + AS1517499 groups, respectively. All rats in the sham-operated group survived. The average SBP in the operated group (196.64 ± 11.07 mmHg) was significantly higher than that in the sham-operated group (104.33 ± 14.92 mmHg) 12 weeks after surgery, but with no significant difference in SBP between the RHRSP, MSC and MSC+ AS1517499 groups. SBP was 201.40 ± 4.88, 198 ± 13.49 and 193.6 ± 15.41 mmHg, respectively, 4 weeks after intervention.

### Cognition Function

**Figure 2:**
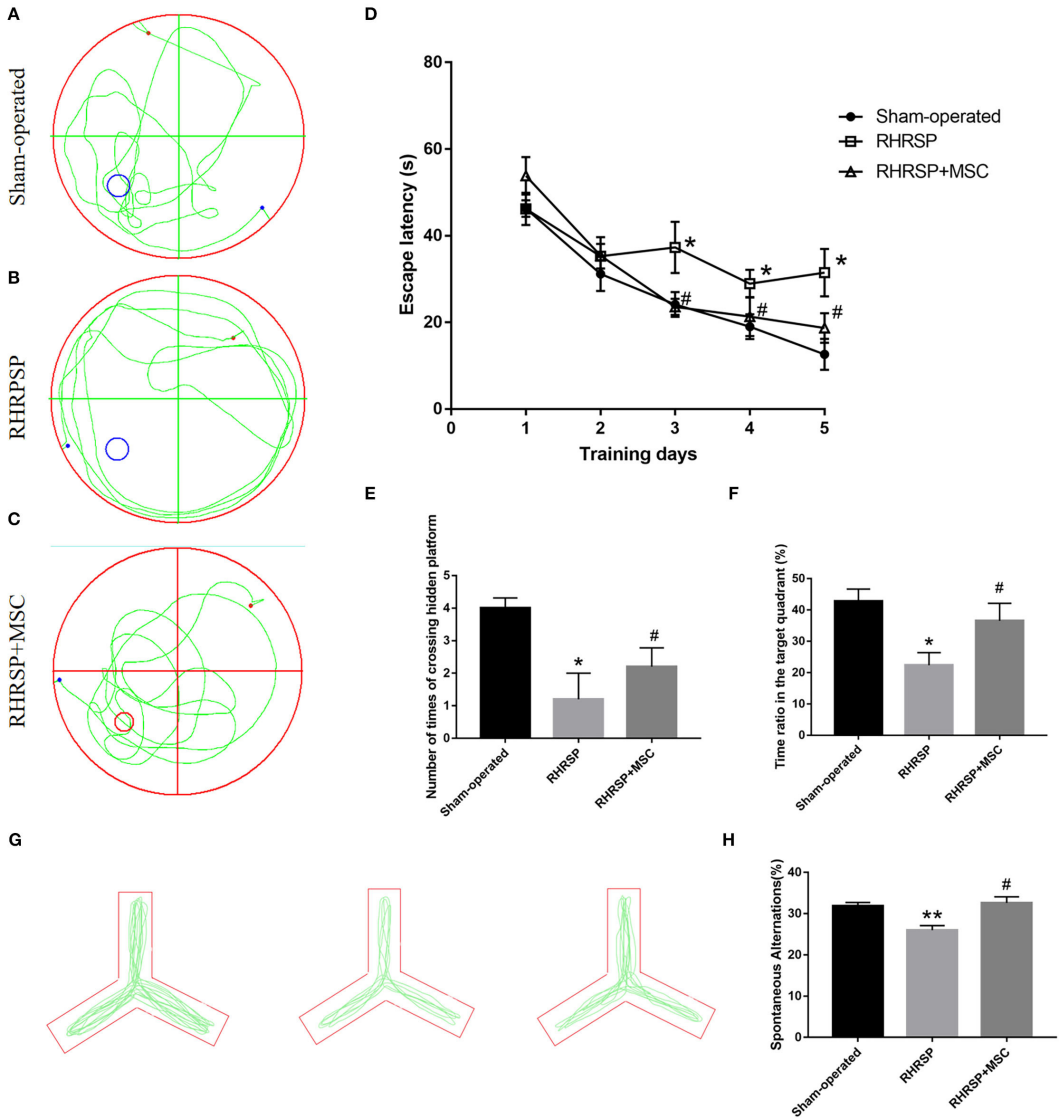
MSCs improved memory, learning, and cognitive behavior in RHRSP rats. **(A–C)** the representative trajectories of spatial probe test at the last day. **(D)** mean escape latency to reach the platform during training days. **(E,F)** the number of times crossing the hidden platform and the time spent in the target quadrant where the platform was previously present. **(G)** the representative trajectories spontaneous alteration behaviors of Y maze. **(H)** the spontaneous alternations of Y maze (*P < 0.05, **P < 0.01, vs. Sham-operated group; #*P* < 0.05, vs. RHRSP group; n = 6/group).

For the Morris water maze test, the escape latencies gradually declined during the training period, but the RHRSP group showed longer escape latencies in the last three training days (Figure 2D). In terms of the time ratio in the target quadrant and the number of times of crossing the hidden platform, the RHRSP rats showed a shorter time ratio and less number of times compared with those in the sham-operated group, suggesting a spatial cognitive disturbance in the RHRSP rats. On the contrary, the rats in the MSC group showed shorter escape latencies, longer time spent in the target quadrant and a greater number of times to cross the hidden platform compared with RHRSP rats, indicating that MSCs improved the cognitive tasks (Figures 2A–C,E,F). The Y-maze data also revealed similar results, in that the RHRSP rats had a reduced percentage of spontaneous alternations than had the rats in the sham-operated group, indicating impaired memory function. However, the percentage of MSC group significantly increased compared with that in the RHRSP group (Figures 2G,H).

### White Matter Lesions

**Figure 3:**
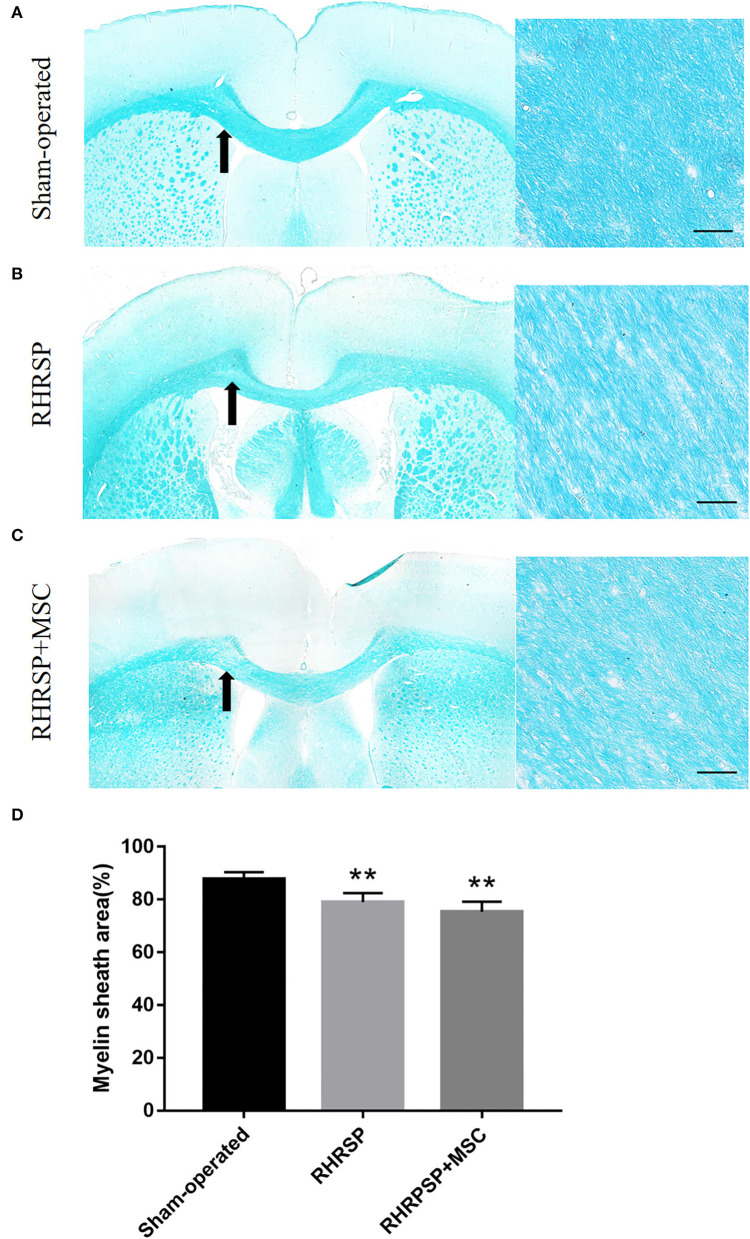
LFB staining of white matter. **(A–C)** Representative images of LFB staining of Sham-operated, RHRSP and RHRSP+MSC groups. Arrows indicate the main observation area. **(D)** Quantification of myelin sheath area (**P < 0.01, vs. Sham-operated group; n = 6/group). scale bar = 50 μm.

As shown in Figures 3A–C, the RHRSP and RHRSP + MSC groups showed demyelination in the corpus callosum that was characterized by vacuole formation and loss of nerve fibers. Figure 3D shows the statistical results of myelin sheath staining area. The RHRSP and RHRSP + MSC groups had less myelin area compared with the sham-operated group, but with no significant difference between the RHRSP and RHRSP + MSC groups.

### Deposition of Aβ1–42

**Figure 4:**
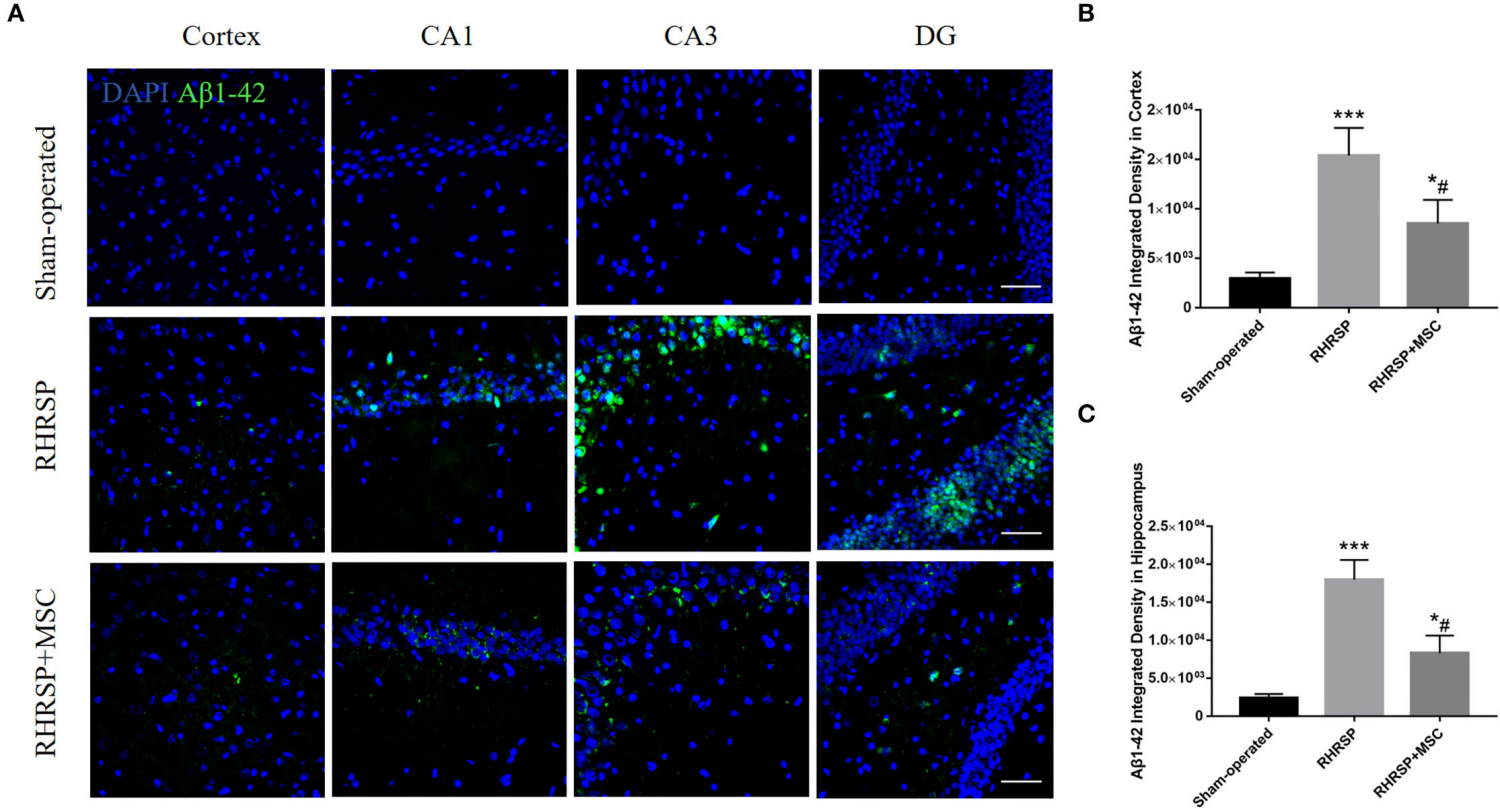
Effects of MSCs on Aβ1-42 accumulation. **(A)** Immunofluorescent staining of Aβ1–42 in three groups. **(B,C)** Quantification of Aβ1–42 fluorescence intensity in the cortex and hippocampus (***P < 0.001, vs. sham-operated group; #*P* < 0.05, vs. RHRSP group; n = 6/group). scale bar = 50 μm. *P < 0.05, vs. sham-operated group.

The Aβ1-42 deposition in the cortex and hippocampus [the average integrated density of the CA1, CA3, and dentate gyrus (DG)] was assessed (Figure 4A). The integrated density of Aβ1–42 deposition in the cortex and hippocampus increased in the RHRSP rats. While in the MSC group, Aβ1–42 deposition in the aforementioned regions was markedly lower than in the RHRSP group (Figures 4B,C).

### Expression and Polarity Distribution of AQP4

**Figure 5:**
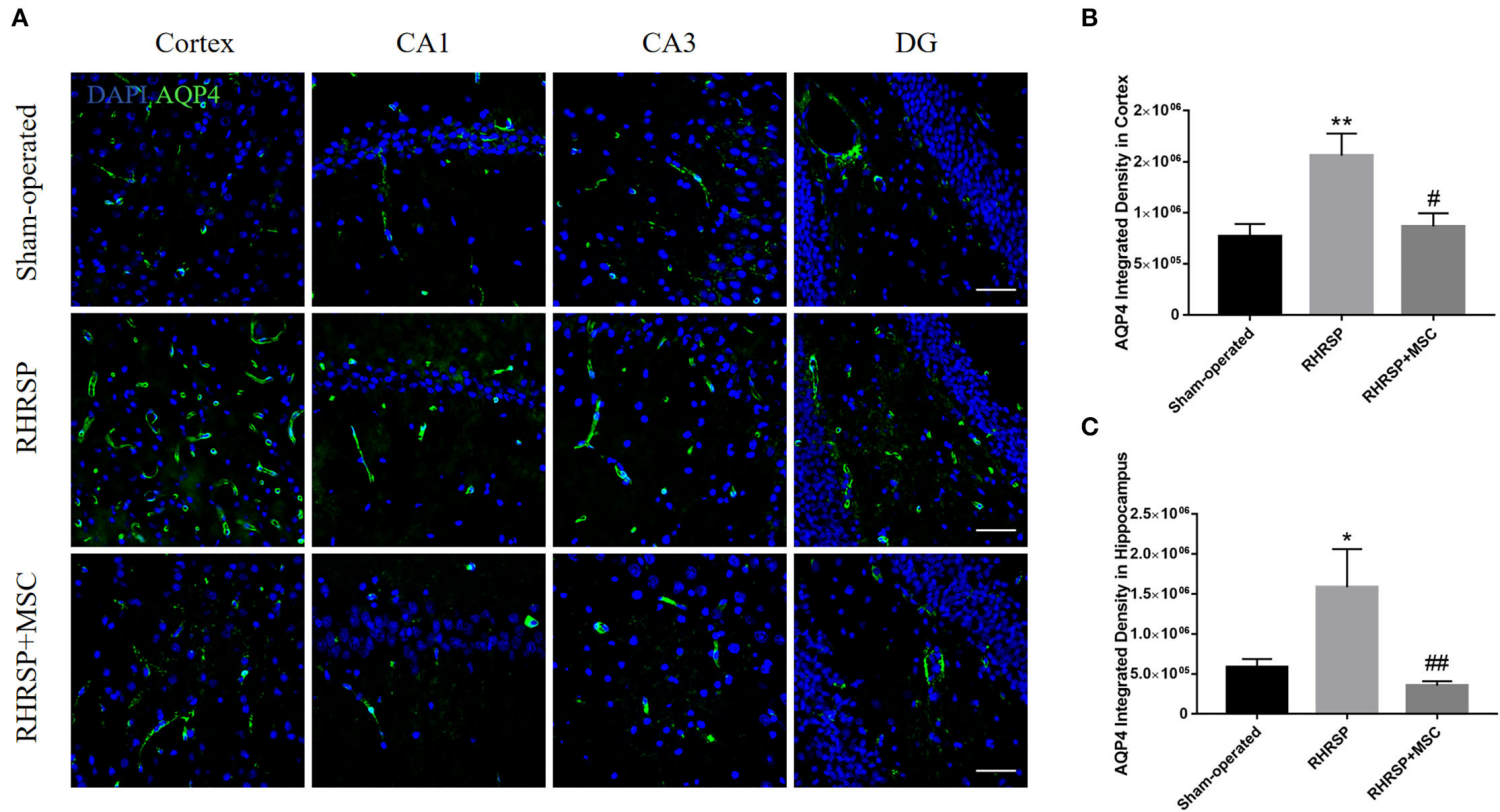
Effects of MSCs on AQP4 expression. **(A)** Immunofluorescent staining of AQP4 in the cortex and hippocampus. **(B,C)** Quantification of the fluorescence intensity of AQP4 in the cortex and hippocampus (*P < 0.05, **P < 0.01, vs. sham-operated group; #*P* < 0.05, ##P < 0.01, vs. RHRSP group; n = 6/group). scale bar = 50 μm.

**Figure 6:**
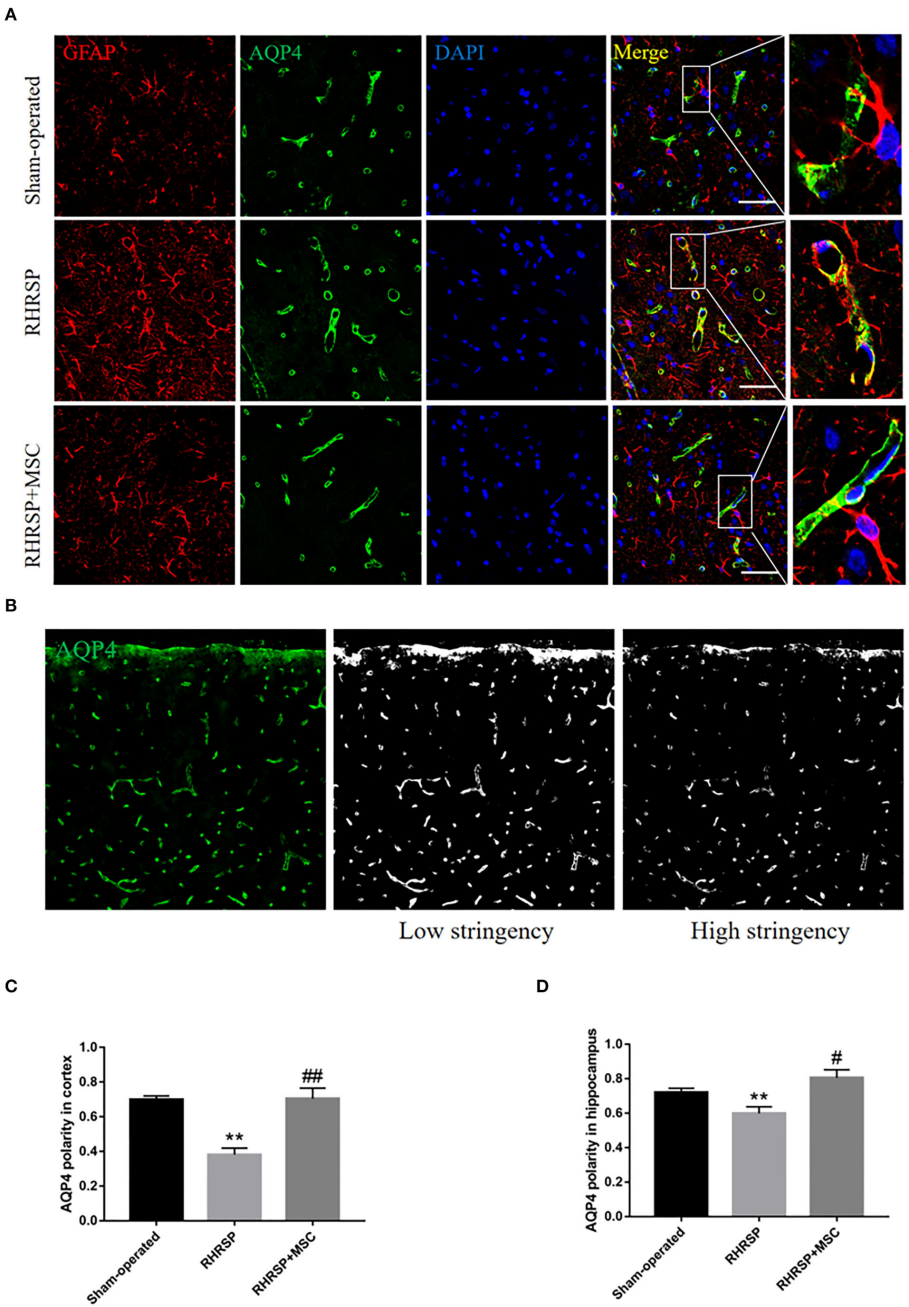
Effects of MSCs on AQP4 polarity. **(A)** Representative images of AQP4 polarity in three groups. **(B)** The sample images of low-stringency and high-stringency of AQP4, thus received the “AQP4 polarity”, which is the ratio of the mean intensity of low-stringency to high-stringency. **(C,D)** Quantification of the AQP4 polarity in the cortex and hippocampus (**P < 0.01, vs. sham-operated group, #*P* < 0.05, ##P < 0.01, vs. RHRSP group; *n* = 6/group). scale bar = 25 μm.

The integrated density of the AQP4 deposits clearly increased in the cortex, CA1, CA3, and DG areas in RHRSP rats compared with the sham-operated group. On the contrary, the RHRSP + MSC group presented a significant reduction in the levels of AQP4 expression (Figure 5). Furthermore, significantly higher expression of AQP4 was found within the astrocytic body, but not in the astrocytic end feet, in the RHRSP group, which was contrary to that in the sham-operated group. However, AQP4 polarity was greatly restored in the MSC group (Figure 6A). Figure 6B shows the sample images of low stringency and high stringency of AQP4. Following the previous methodology, the“AQP4 polarity” was calculated using the ratio of the mean intensity of low stringency to high stringency. Figures 6C,D shows a higher AQP4 polarity after MSC treatment in the cortex and hippocampus than in the RHRSP group.

### Activation of Microglia and Astrocytes

**Figure 7:**
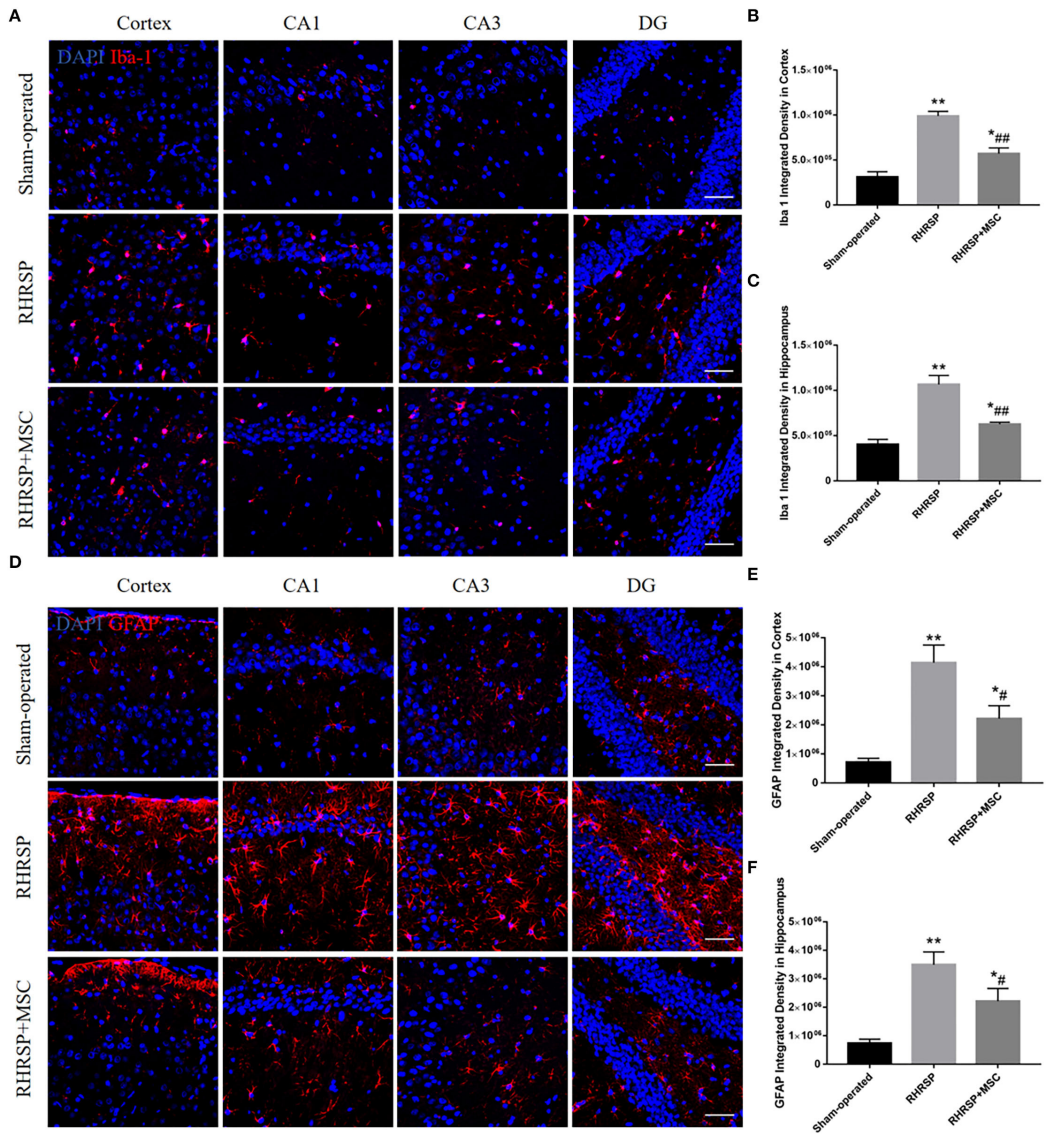
Effects of MSCs treatment on the activation of microglia and astrocytes. **(A,D)** Representative images of immunofluorescence staining of Iba-1 and GFAP in the cortex and hippocampus. **(B,C,E,F)** Quantification of immunofluorescence staining for Iba-1 and GFPAP in the cortex and hippocampus (*P < 0.05, **P < 0.01, vs. sham-operated group; #*P* < 0.05, ##*P* < 0.01, vs. RHRSP group; n = 6/group). scale bar = 50 μm.

Figure 7 shows the activation state of microglia and astrocytes in the three groups. Obvious activation and proliferation of the microglia and astrocytes in the cortex and hippocampus were found in the RHRSP group rats compared with those in the sham-operated group rats, treatment with MSCs inhibited the aforementioned reaction.

### Phenotype Distribution of Microglia and Astrocytes

**Figure 8:**
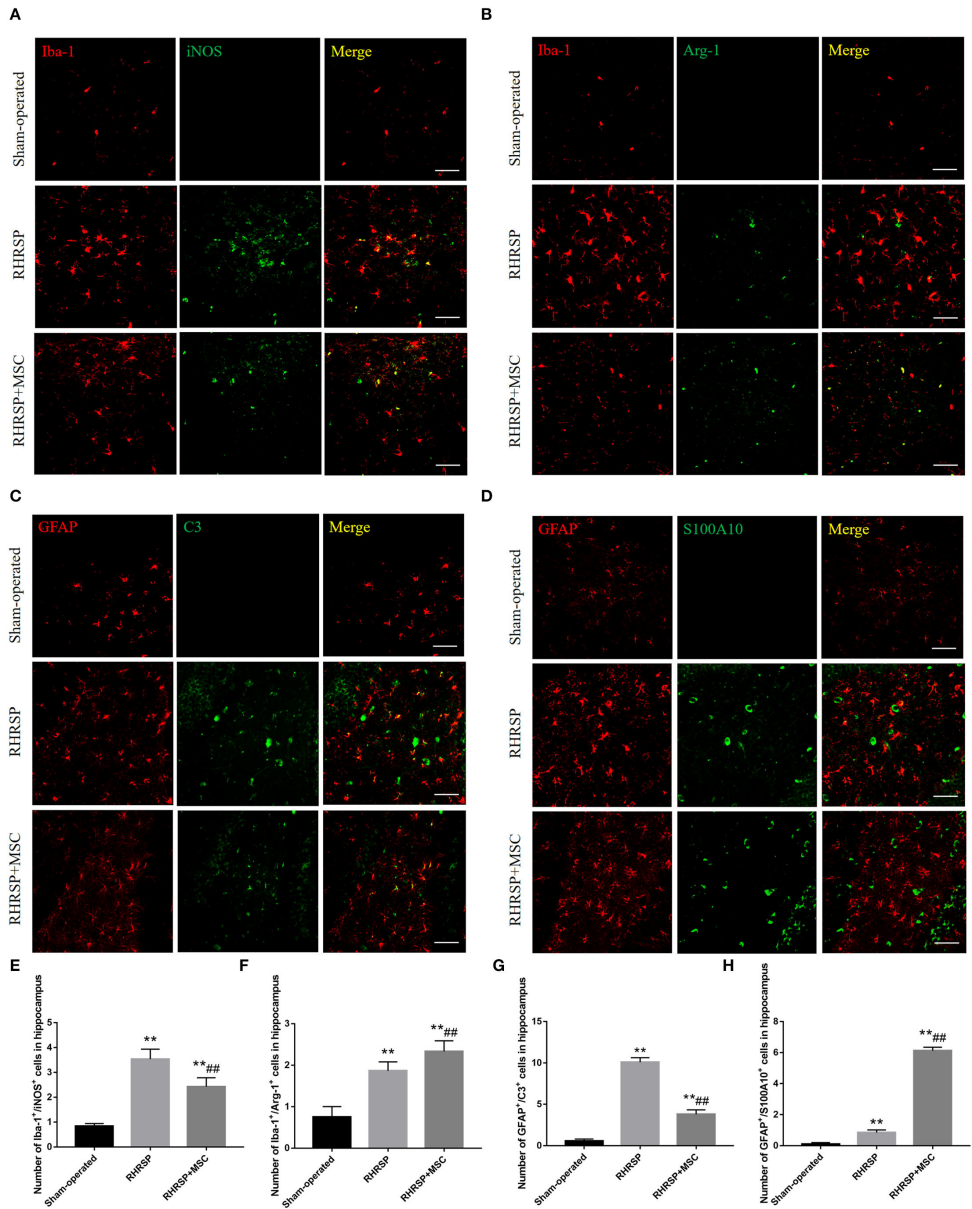
Effects of MSCs treatment on the phenotype distribution of microglia and astrocytes. **(A–D)** Double immunofluorescence staining of Iba-1/iNOS-positive M1 microglia, Iba-1/Arg-1-positive M2 microglia, GFAP/C3-positive A1 astrocytes and GFAP/S100A10-positive A2 astrocytes in hippocampus. **(E–H)** Representative quantification showing that MSC significantly decreased the number of M1, A1 cells and increased the number of M2, A2 cells in hippocampus. (**P < 0.01, vs. Sham-operated group, ##*P* < 0.01, vs. RHRSP group; *n* = 6/group). scale bar = 50 μm.

The study next explored the presence of M1 microglia (Iba-1/iNOS positive cells) and M2 microglia (Iba-1/Arg-1 positive cells) in the hippocampus and found that MSC- treated rats had significantly fewer Iba-1/iNOS -positive cells (Figures 8A,E) and more Iba-1/Arg-1-positive cells than RHRSP rats (Figures 8B,F). Also, C3-positive astrocytes (a marker of A1 astrocytes) were obviously fewer in the RHRSP + MSC group than in the RHRSP group (Figures 8C,G). On the contrary, the RHRSP rats administered MSCs had a significantly higher number of S100A10-positive astrocytes (a marker of A2 astrocytes) compared with the RHRSP rats (Figures 8D,H).

### Expression of Cytokines

**Figure 9:**
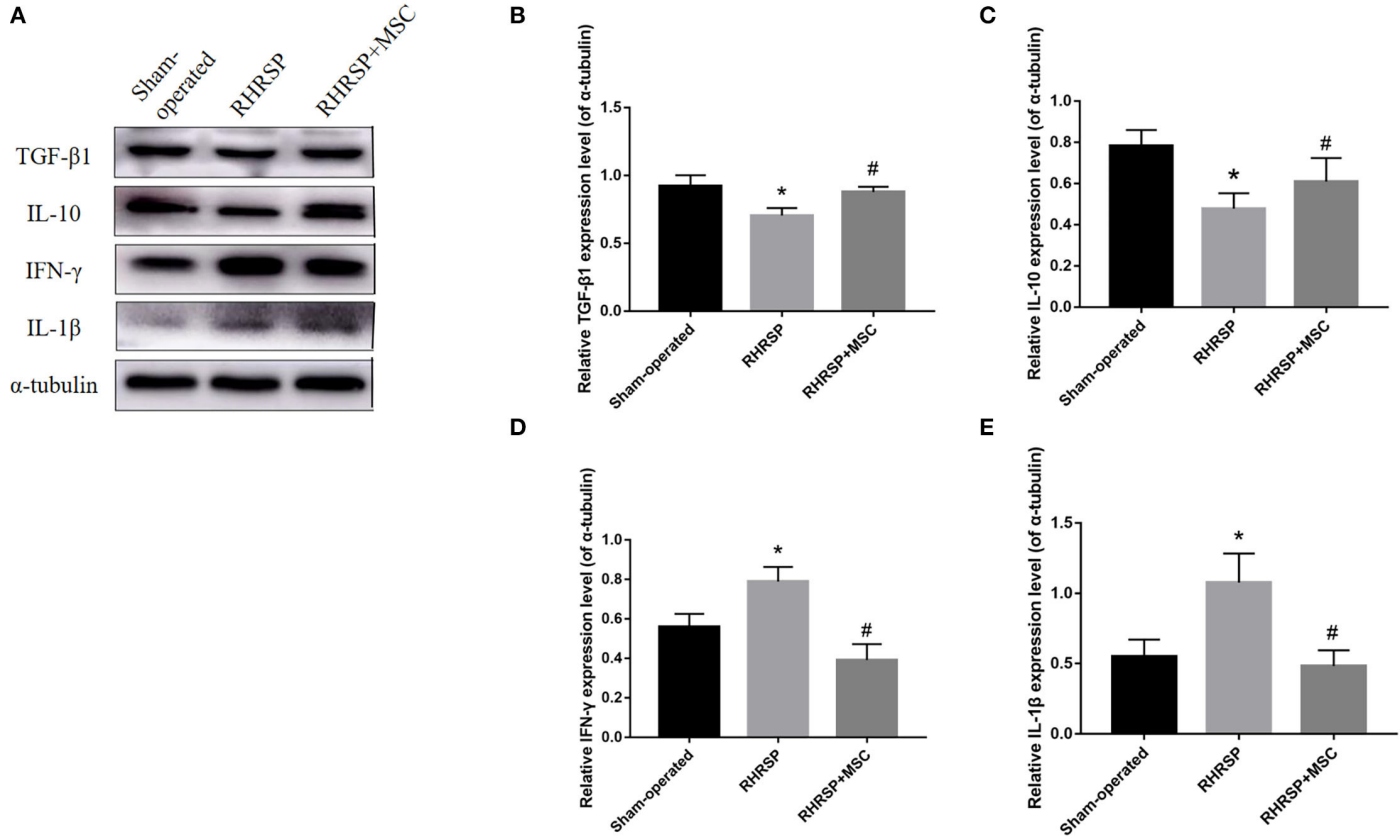
Effects of MSCs treatment on the expression of cytokines in the hippocampus. **(A)** Representative images of western blot analysis of TGF-β1, IL-10, IFN-γ, IL-1β and α-tubulin in the hippocampus in three groups. **(B–E)** Quantification of the relative protein expression levels of TGF-β1, IL-10, IFN-γ, IL-1β. (*P < 0.05, vs. sham-operated group, #P < 0.05, vs. RHRSP group; n = 6/group).

Figure 9 shows the expression of inflammatory and anti-inflammatory mediators derived from M1/M2 microglia in the hippocampus. Higher expression of the inflammatory mediators IL-1β and IFN-γ and lower expression of anti-inflammatory mediators TGF-β1 and IL-10 were found in RHRSP group, while the two kinds of factors were down regulated and up regulated, respectively, following MSCs treatment.

### Expression of pSTAT6 and Cytokines After AS1517499 Intervention

**Figure 10:**
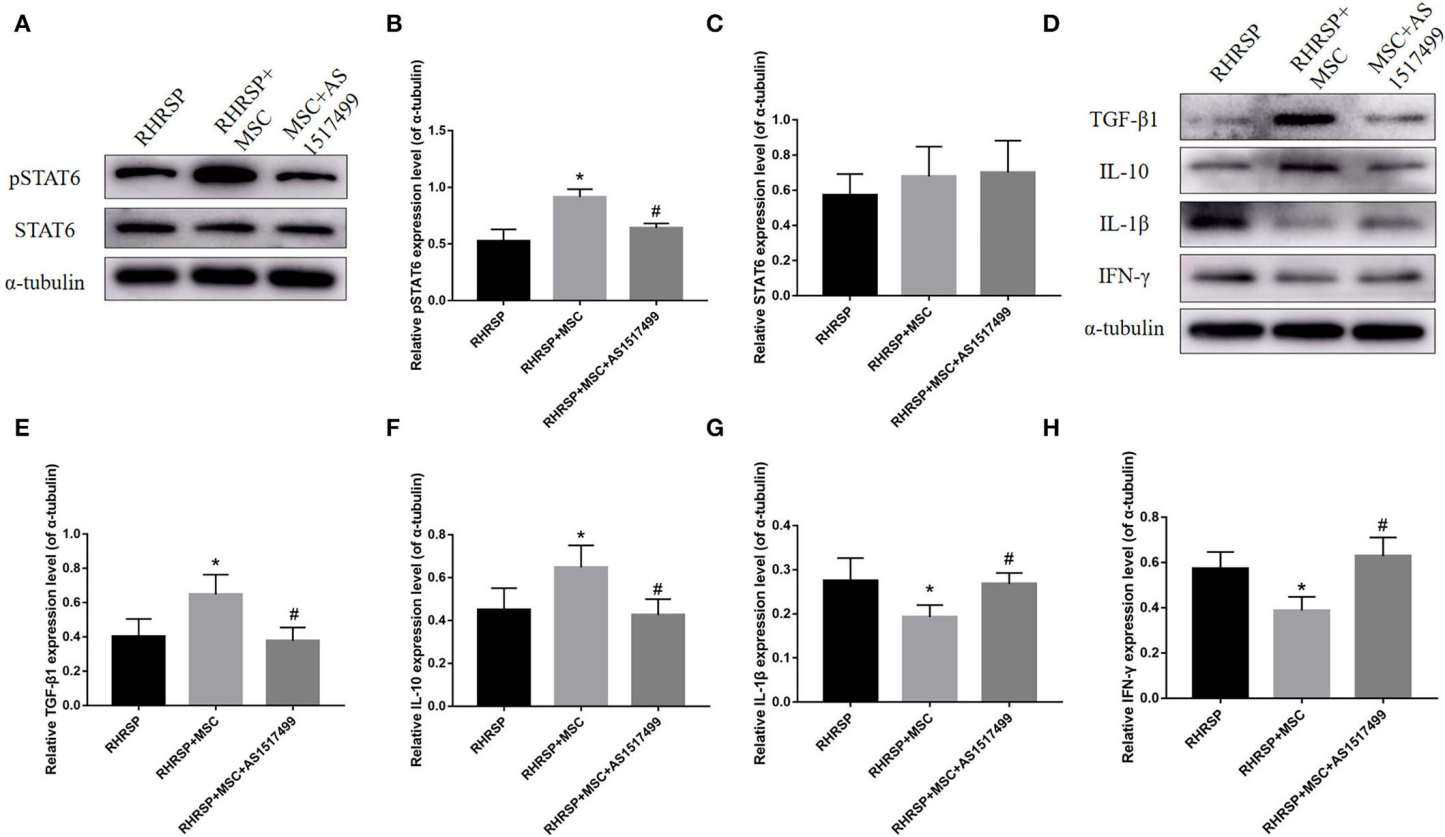
Effects of AS1517499 on the expression of STAT6, pSTAT6 and cytokines in the hippocampus after AS1517499 acquisition. **(A)** Representative images of western blot analysis of STAT6, pSTAT6 and α-tubulin. **(B,C)** Quantification of the relative protein expression levels of STAT6 and pSTAT6. **(D)** Representative images of western blot analysis of cytokines in the hippocampus in three groups after AS1517499 acquisition. **(E–H)** Quantification of the relative protein expression levels of TGF-β1, IL-10, IL-1β and IFN-γ. (*P < 0.05, vs. sham-operated group, #*P* < 0.05, vs. RHRSP group; *n* = 6/group).

The study also evaluated M1- and M2-related inflammatory and anti-inflammatory mediators in the RHRSP, MSC, and MSC + AS1517499 groups. The MSC group showed a significant increase in pSTAT6 expression compared with the RHRSP group, and the MSC + AS1517499 inhibited the increase in pSTAT6 caused by MSCs. Moreover, MSC + AS1517499 reversed the increase in the levels of M2-related anti-inflammatory cytokines and the decrease in the levels of M1-related anti-inflammatory cytokines regulated by MSCs (Figure 10).

## Discussion

The present study used RHRSP rats to determine the effects of MSCs on the cognitive impairment and Aβ deposition in the brain of CSVD. In the RHRSP rats, significant spatial cognitive impairment, white matter lesions (WMLs) and Aβ deposition were detected. MSCs effectively improved the cognition and reduced Aβ deposition despite no improvement in WMLs. MSCs also significantly restored AQP4 polarity and alleviated neuroinflammation by regulating the phenotypic distribution of glial cells. Therefore, this study provided evidence of the effectiveness of MSCs against plaque deposition and cognitive impairment CSVD rats, suggesting the potential therapeutic effect of MSCs on CSVD.

Aβ deposition is one of the important causes of cognitive decline and is associated with neurodegenerative diseases, especially AD. It is increasingly found to be associated with age-related cerebral vascular diseases. Previous studies proposed that cerebrovascular injury was a major risk factor for AD dementia (Arvanitakis et al., 2016). It has been suggested that the dysfunction of efflux transport at the BBB causes the accumulation of Aβ in AD, leading to progressive cognitive dysfunction. On the other hand, influx of neurotoxic cell fragments results in inflammatory responses (van Assema et al., 2012). CSVD is a kind of cerebrovascular disease highly correlated with aging. Besides vascular Aβ lesions found in CAA, an association between Aβ and CSVD has been reported in many studies. Liu et al. found that serum Aβ42 combined with the CSVD score could predict cognitive impairment in patients with CSVD (Liu et al., 2021). An 8-year longitudinal study suggested that Aβ positivity in amyloid PET was an independent predictor of functional disability regardless of the occurrence of stroke in patients with subcortical VCI (Kang et al., 2022). The present study showed the deposition of Aβ in the brain of rats with CSVD, which was consistent with clinical findings. Many studies suggested that the intravenously injected stem cells could cross the blood-brain barrier and migrate to the lesion, thus inducing nerve regeneration, re-myelination and immune regulation. In particular, they reduced neuroinflammation and Aβ deposition (Urdzíková et al., 2014; Lykhmus et al., 2019). This study found that MSCs reduced Aβ deposition, thus providing new ideas that MSC intervention could alleviate Aβ deposition and cognitive impairment in CSVD.

Although Aβ decreased after treatment, no significant improvement was found in WMLs. The pathogenesis of WMLs is still unclear. The most possible pathogenic factor is BBB destruction; in addition, there are oxidative stress, immune inflammation, and impaired cerebral blood flow automatic regulation (Lin et al., 2017). The permeation of toxic substances including Aβ may also be involved in the formation of WMLs (Zhang et al., 2019; McAleese et al., 2021). At present, the temporal and spatial changes in Aβ and WML expression in the brain and the mechanism of promoting cognitive decline are rarely reported. The present study found that MSC treatment reversed Aβ deposition, but not WMLs, which indirectly explained the complexity of the formation of WMLs. Thus, it was hypothesized that the aggregation of Aβ was an important segment in the development of WMLs, but the formation of WMLs might be the result of a combination of many other factors and predated Aβ aggregation. This finding suggested that the dynamic change in WMLs and Aβ deposition should be observed in future studies.

Aβ accumulation in the brain is due to the imbalance between its production and clearance. Aβ clearance has been thought to be associated with the dysfunction of glymphatic system (Rasmussen et al., 2018). The glymphatic system in the brain plays an indispensable role in clearing waste products, and is responsible for a substantial part of the total Aβ clearance during sleep (Xie et al., 2013). The paravascular material exchange, mainly through cerebrospinal fluid (CSF) and interstitial fluid (ISF), is supported by the astrocytes involving AQP4 (Kress et al., 2014), which normally localizes to the perivascular astrocytic end feet. The knockout of AQP4 is known to significantly affect the clearance process, which gets aggravated when accompanied by neuroinflammation and the failure of perivascular AQP4 polarization (Iliff et al., 2012). The polarity of AQP4 refers to AQP4 located in the astrocyte foot, but astrocytes dysfunction, such as activation, leads to AQP4 redistribution to the astrocyte body. As a result, the clearance of brain metabolic waste gets blocked (Heppner et al., 2015). The present study showed thatAQP4 polarization was lost, and more AQP4 was redistributed from the foot processes to the cell body in CSVD rats. However, AQP4 expression significantly increased in the brains of RHRSP rats, which was consistent with the findings of several studies related to chronic cerebral hypoperfusion. It was probably related to astrocyte edema after ischemia (Manley et al., 2000). This study demonstrated that MSCs effectively downregulated AQP4 expression and restored AQP4 polarization, thus relieving cerebral edema and promoting Aβ clearance.

Neuroinflammation, known as glial cell activation, releases inflammatory cytokines that are thought to be partly responsible for cognitive impairment (Singh and Abraham, 2017). It usually begins with the activation of microglia and is considered to be closely associated with neurodegenerative disorders such as AD, PD, and multiple sclerosis (Lucin and Wyss-Coray, 2009; Huang et al., 2015; Subedi et al., 2017; Li et al., 2019; Thawkar and Kaur, 2019). It is usually accompanied by Aβ deposition because Aβ can activate microglia. Studies have shown that Aβ42 can increase the expression of M1-type markers and decrease the expression of M2-type markers in microglia cultured *in vitro*(Cheyuo et al., 2012). Meanwhile, the activated glial cells secrete a variety of inflammatory factors, which cause tissue damage and further aggravate the accumulation of Aβ. Thus, the inhibition of inflammatory response can relieve the severity of such neurodegenerative diseases (Vlad et al., 2008). Later, Rosenberg (2017) put forward that chronic hypertension could lead to neuroinflammation. The present study revealed the phenomenon in the CSVD rats as well. Activated microglia consists of two types: M1 and M2 (Allendorf et al., 2020). M1 microglia produces various pro-inflammatory factors, such as IL-1β, TNF-α, and IFN-γ, and promotes the inflammatory process (Gupta et al., 2018). However, M2 microglia release anti-inflammatory cytokines, such as IL-10 and TGF-β, engulf damaged cell fragments, and promote tissue restoration and neuron regeneration (Liu et al., 2019). In analogy with the “M1”/ “M2” microglia(Martinez and Gordon, 2014; Heppner et al., 2015), two types of activated astrocytes exist, which are termed “A1” (neurotoxic phenotype) and “A2” (neuroprotective phenotype), respectively in the case of neuroinflammation. Importantly, a recent study uncovered the decisive effect of reactive microglia on astrocyte polarization (Liddelow et al., 2017), confirming that the neurotoxic phenotype, A1 astrocytes were motivated by activated neuroinflammatory microglia. Similarly, A1 astrocytes significantly upregulated the intensity of complement cascade shown to be destructive to synapses, while A2 astrocytes upregulated many neurotrophic factors. In this study, the infusion of MSCs significantly attenuated the activation of microglia and astrocytes. Furthermore, it downregulated the M1/A1 phenotype and upregulated the M2/A2 phenotype in RHRSP rats, reduced inflammatory cytokine production that was M1-related and promoted the expression of M2-related anti-inflammatory cytokines. The possible reasons were that the reduction of MSC-induced Aβ reduced the conversion of microglia into M1, and MSCs might also be directly involved in regulating the glial cell phenotype.

The STAT family is involved in regulating the functional status of microglia/macrophages; the STAT6 signaling pathway appears to induce the transformation of macrophages into an anti-inflammatory phenotype (Brunn et al., 2014; Hu et al., 2015). The knockout of STAT6 has been found to result in the dysfunction of dead neuron clearance and increased expression of pro-inflammatory factors in mice with cerebral ischemia (Cai et al., 2019). Thus, the present study investigated the relationship between inflammatory cytokines and pSTAT6 to explore the mechanism underlying the neural immunomodulatory functions of MSCs. We discovered that MSCs could regulate the expression of inflammatory cytokines by activating the STAT6 signaling pathway while the pharmacological inhibitor of pSTAT6 (AS1517499) suppressed the effect, suggesting that pSTAT6 played a crucial role in the immunomodulatory process of MSCs.

In conclusion, this study showed the cognitive impairment and Aβ deposition in CSVD rats. MSC treatment improved the cognition and reduced Aβ accumulation despite no WML improvement, possibly in part through restoration of AQP4 polarity and alleviation of neuroinflammation. Furthermore, MSCs might regulate the microglia phenotype by activating the STAT6 signaling pathway to further relieve neuroinflammation.

A limitation of this study was that it used young rather than aged animals. The cognitive impairment and neuroimaging and pathological changes, except the aging of human CSVD, were better simulated in the chronic hypertensive rats (Fan Y. L. et al., 2015; Lan et al., 2015). Aging is an independent risk factor for stroke, and studies suggested that the self-regulation of cerebral blood flow might be altered with age (Wollner et al., 1979; Lartaud et al., 1993). Moreover, the incidence and imaging markers of CSVD increased with aging (Mu et al., 2022). Despite its importance, it is difficult to replicate the factor in rats because of their harder feeding and longer life span compared with mice. Therefore, it has always been a difficult problem in the animal research of CSVD. We will explore a more appropriate animal model of CSVD combined with the factor of aging in future studies.

## Data Availability Statement

The original contributions presented in the study are included in the article/supplementary material, further inquiries can be directed to the corresponding authors.

## Ethics Statement

The animal study was reviewed and approved by the Animal Research Ethics Committee at Sun Yat-sen University.

## Author Contributions

XL, ZP, and YF designed the research. XL, FO, LH, PS, JY, YS, ML, and LL performed the experiments. XL drafted the manuscript. All authors approved the final version of the manuscript.

## Funding

This study was supported by the grants from the Guangzhou Science and Technology Program Key Project (No. 202007030010), National Natural Science Foundation of China (No. 82071294), Guangzhou Municipal Science and Technology Project (No. 202002030073), Guangdong Provincial Key Laboratory of Diagnosis and Treatment of Major Neurological Diseases (No. 2020B1212060017), Guangdong Provincial Clinical Research Center for Neurological Diseases (No. 2020B1111170002), the Southern China International Cooperation Base for Early Intervention and Functional Rehabilitation of Neurological Diseases (Nos. 2015B050501003 and 2020A0505020004), and Guangdong Provincial Engineering Center for Major Neurological Disease Treatment, and Guangdong Provincial Translational Medicine Innovation Platform for Diagnosis and Treatment of Major Neurological Disease.

## Conflict of Interest

The authors declare that the research was conducted in the absence of any commercial or financial relationships that could be construed as a potential conflict of interest.

## Publisher's Note

All claims expressed in this article are solely those of the authors and do not necessarily represent those of their affiliated organizations, or those of the publisher, the editors and the reviewers. Any product that may be evaluated in this article, or claim that may be made by its manufacturer, is not guaranteed or endorsed by the publisher.
